# Isolated cuboid dislocation: case report

**DOI:** 10.1093/jscr/rjae563

**Published:** 2024-08-30

**Authors:** Amaurilio de Oliveira Lima, Antonio Bezerra de Albuquerque Filho, Gustavo Henrique Carillo Ambrosio, Leonardo Vasconcelos Coelho, Vitor Yoshiura Massuda, Paulo Henrique Schmidt Lara

**Affiliations:** Hospital Municipal Prefeito Edvaldo Orsi – Ouro Verde, Avenue Ruy Rodriguez, 3434 - Chácara São José, Campinas - SP, 13060-646, Brazil; UNIFESP – Escola Paulista de Medicina – SP – Street Sena Madureira, 1500 - Vila Clementino, São Paulo - SP, 04021-001, Brazil; UNIFESP – Escola Paulista de Medicina – SP – Street Sena Madureira, 1500 - Vila Clementino, São Paulo - SP, 04021-001, Brazil; Conjunto Hospitalar do Mandaqui, Street Voluntários da Pátria, 4301 - Santana, São Paulo - SP, 02401-400, Brazil; UNIFESP – Escola Paulista de Medicina – SP – Street Sena Madureira, 1500 - Vila Clementino, São Paulo - SP, 04021-001, Brazil; UNIFESP – Escola Paulista de Medicina – SP – Street Sena Madureira, 1500 - Vila Clementino, São Paulo - SP, 04021-001, Brazil

**Keywords:** cuboid, joint dislocations, midfoot, lateral column, case reports

## Abstract

Isolated cuboid dislocation without fracture is a rare injury, and there is a lack of literature describing its treatment. Studies report the use of closed or open reduction, with Kirschner wire fixation in the treatment of these injuries. This case report presents the clinical condition of a 24-year-old male patient who arrived at the emergency department with an isolated dislocation of the right foot cuboid bone without the presence of a fracture, after suffering trauma during a football game. Open reduction was performed in the surgical center with stabilization and fixation using a Kirschner wires. The patient showed an excellent response to the treatment, with no loss of the foot’s range of motion.

## Introduction

Cuboid dislocations are rare and difficult-to-diagnose injuries. It is an injury with few recorded cases and few published studies on the subject [1].

The proposed mechanism of injury includes forced inversion and plantar flexion movement of the foot, with a medial plantar force allowing the cuboid to dislocate below the calcaneus. This patient presented with an isolated inferomedial cuboid dislocation and underwent open reduction and internal fixation with a Kirschner wire. Physical examination and simple radiographs were used for diagnosis.

## Case report

A healthy 24-year-old patient, without comorbidities, physically active, with a body mass index of 24 kg/m^2^, suffered trauma during a football game, resulting in an inversion twist of the right foot. He experienced intense pain at the moment of trauma, forcing him to stop participating in the football event. He arrived at the emergency room reporting severe pain in the lateral region of the right midfoot.

**Figure 1:**
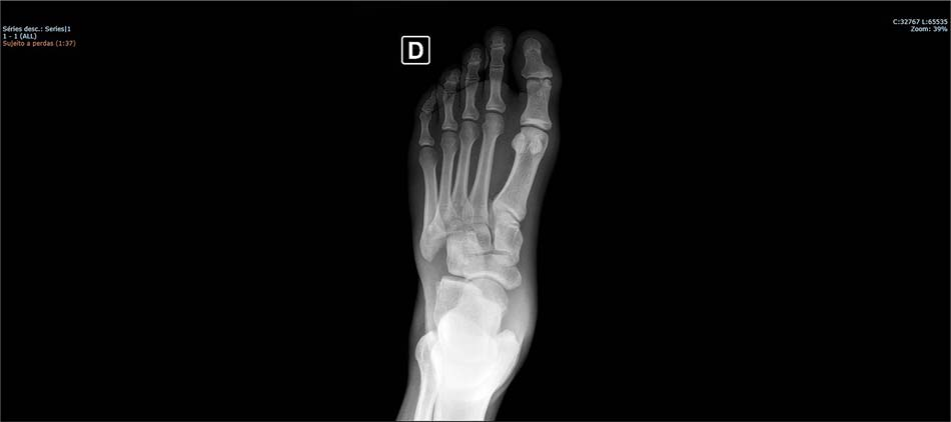
Preoperative X-ray of the foot in AP view

**Figure 2:**
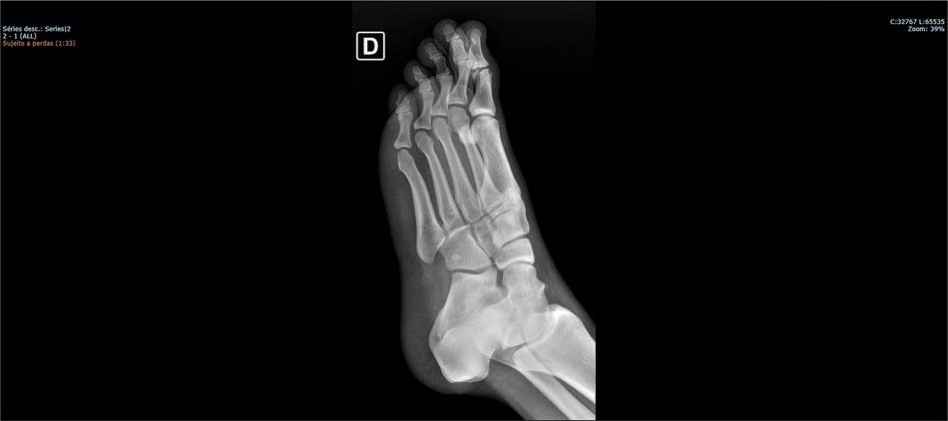
Preoperative X-ray of the foot in lateral view

On physical examination, there was pain upon palpation over the dorso-lateral region of the midfoot, mild edema, hyperemia, inability to walk, and restricted ankle extension. Based on the physical examination, radiographs were requested. The examinations (Figs 1 and 2) showed inferomedial displacement of the cuboid bone without the presence of a fracture, diagnosing an isolated cuboid dislocation. He was immobilized and admitted for an open surgical reduction procedure.

**Figure 3:**
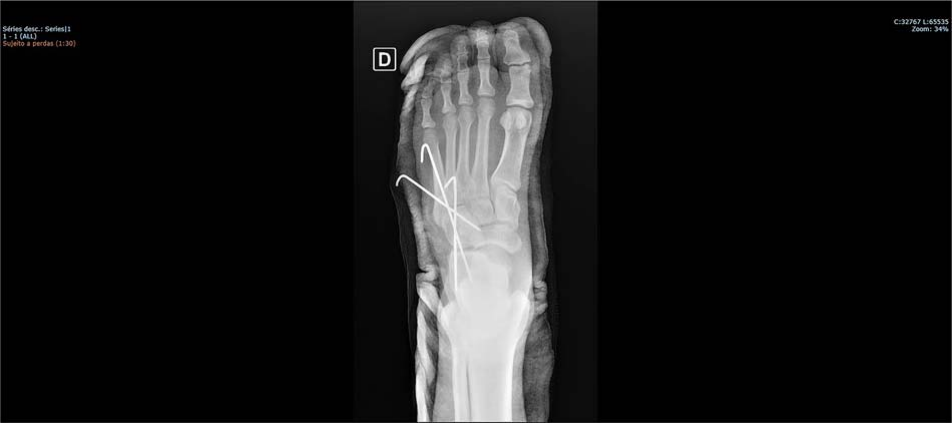
Postoperative X-ray of the foot in AP view

**Figure 4:**
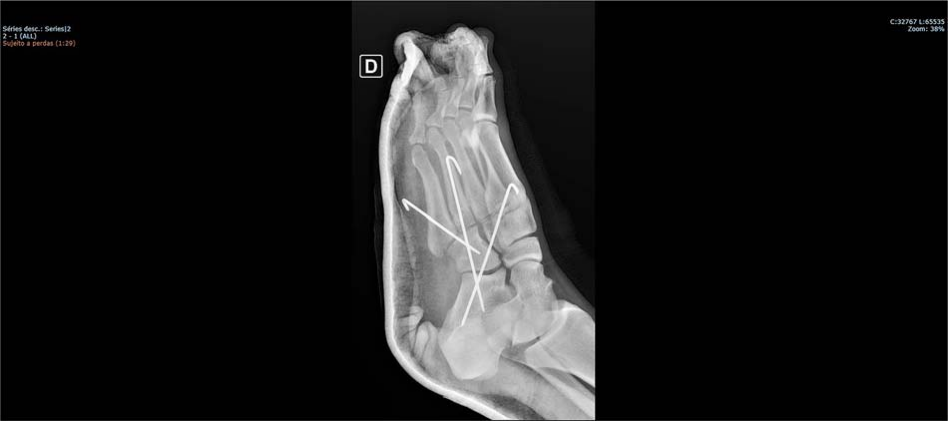
Postoperative X-ray of the foot in lateral view

The patient was placed on the surgical table in the supine position, anesthetized with spinal anesthesia, and given prophylactic antibiotic therapy with 2 g of cefazolin. After exsanguination of the lower limb, a dorsolateral incision was made on the foot over the cuboid bone and deepened through the layers until complete visualization of the cuboid bone was achieved. There was an interposition of ligamentous structures, such as the calcaneocuboid ligament, dorsal cuneocuboid ligament, and dorsal tarsometatarsal ligaments. After removing all structures that were interposed and preventing reduction, the cuboid was reduced easily, but instability was observed. Due to the instability found, percutaneous fixation was performed with three Kirschner wires: the first extending from the fourth metatarsal to the calcaneus, traversing the cuboid; the second from the fifth metatarsal to the cuboid; and the third from the cuboid to the calcaneus, from anterior-dorsal to posterior-plantar (Figs 3 and 4). After complete fixation, the stability of the cuboid was observed, maintaining it in the correct position, confirmed with fluoroscopy during surgery.

The patient was instructed not to bear weight until being re-evaluated two weeks postoperatively, at which point the sutures were removed, and no complications with the wound were observed. At the second follow-up appointment 6 weeks later, the Kirschner wires were removed, weight-bearing was allowed, and the patient was referred to physical therapy. At the third follow-up appointment, 180 days after surgery, the patient showed a slight decrease in ankle extension, but no pain during walking and running. He experienced no complications and returned to playing football.

In this evaluation, Visual Analog Scale (EVA) and American Orthophaedic Foot and Ankle Society (AOFAS) pain scores were calculated. On the EVA pain scale, he scored zero during daily activities, and on the AOFAS scale, he scored ninety points, demonstrating adequate pain control and functionality.

## Discussion

The cuboid is a multiarticular bone and the most lateral bone in the distal row of the tarsal bones. It is located proximally to the 4th and 5th metatarsal bones, distally to the calcaneus, and laterally to the navicular and cuneiform bones. It contributes to the formation of the following joints: tarsometatarsal, calcaneocuboid, cuboidonavicular, and cuneocuboid. Due to its lateral position, the cuboid bone only contributes to the formation of the lateral longitudinal arch of the foot. It has multiligament support.

Cuboid injury is rare and can be overlooked in the emergency room, being confused with other diagnoses such as ankle sprain. Due to its rarity, there is no consensus on its treatment, with open fixation using Kirschner wires being used in the few reported cases, indicating a need for further studies to introduce new surgical techniques. The treatment performed on the patient with Kirschner wires proved to be successful [1].

Comparing this case with others found in the literature, it was observed that physical examination is of fundamental importance for the diagnostic hypothesis, and that the use of simple radiographs in two views is sufficient for diagnosis, with CT scans helping to elucidate the diagnosis and in surgical planning [2–5]. It was not possible to perform reduction in the emergency room in any case. It was also found that open reduction and percutaneous fixation were successful choices [2–5]. It was concluded that maintaining the Kirschner wires for 6 weeks was the most commonly used duration in other cases [2–5].

## Conclusion

Performing open reduction and percutaneous fixation with Kirschner wire in the emergency setting, and maintaining non-weight-bearing for 6 weeks proved to be satisfactory with positive results for the patient. This approach can be replicated in other cases that may arise in the emergency room.

## Conflict of interest statement

None of the other authors have conflicts of interest to declare.

## References

[1] Jacobson FS . Disloca on of the cuboid. Orthopaedics 1990;13:1387–9. 10.3928/0147-7447-19901201-12 2274483

[2] Sheahan K , Pomeroy E, Bayer TA. An isolated cuboid dislocation. A case report. Int J Surg Case Rep 2017;39:1–4. 10.1016/j.ijscr.2017.06.052. 28779701 PMC5544476

[3] Cooperman S , Derner BS, Rao NM, et al. A rare case of an isolated traumatic cuboid dislocation treated with a novel technique. Foot Ankle Surgs 2023;3:100298–8.

[4] Loyst RA , Hance F, Paulus M. Closed reduction and percutaneous pinning in isolated cuboid dislocation management: a case report. Curēus 2023;15:e49023. 10.7759/cureus.49023. PMC10727692 38111424

[5] Smith JS , Flemister AS. Complete cuboid dislocation in a professional baseball player. Am J Sports Med 2006;34:21–3. 16170045 10.1177/0363546505275012

